# Active learning through discussion: ICAP framework for education in health professions

**DOI:** 10.1186/s12909-019-1901-7

**Published:** 2019-12-30

**Authors:** Jaeseo Lim, Hyunwoong Ko, Ji Won Yang, Songeui Kim, Seunghee Lee, Myung-Sun Chun, Jungjoon Ihm, Jooyong Park

**Affiliations:** 1 Interdisciplinary Program in Cognitive Science, Seoul National University, Seoul, South Korea; 2 Department of Psychology, Seoul National University, Seoul, South Korea; 3 School of Dentistry, Biomedical Knowledge Engineering Laboratory, Seoul National University, Seoul, South Korea; 4 Department of Medical Education, College of Medicine, Seoul National University, Seoul, South Korea; 5 Research Institute for Veterinary Science, Seoul National University College of Veterinary Medicine, Seoul, South Korea; 6 School of Dentistry, Dental Research Institute, Seoul National University, Seoul, South Korea

**Keywords:** Learning outcomes, Discussion, Question generation, Self-study, Medical education

## Abstract

**Background:**

The ICAP framework based on cognitive science posits four modes of cognitive engagement: Interactive, Constructive, Active, and Passive. Focusing on the wide applicability of discussion as interactive engagement in medical education, we investigated the effect of discussion when it was preceded by self-study and further investigated the effect of generating questions before discussions.

**Methods:**

This study was conducted in the second semester of 2018 and was participated in by 129 students majoring in health professions, including medicine, dentistry, veterinary medicine, and nursing. The students were assigned to four different trial groups and were asked to fill out a Subjective Mental Effort Questionnaire after completing each session. Their performance in posttest scores was analyzed using Bonferroni test, and mental effort was analyzed using mediation analysis.

**Results:**

These results indicated that the self-study and question group had the highest performance and that the lecture and summary group had the lowest performance when comparing the total score. Using the analysis of mental effort, it was confirmed that the relationship between different study conditions and post-test performance was mediated by mental effort during test.

**Conclusions:**

Our findings support the ICAP framework and provide practical implications for medical education, representing the fact that students learn more when they are involved in active learning activities, such as self-study and question generation, prior to discussions.

## Background

A large number of university students majoring in health professions have achieved outstanding academic performances. Nonetheless, they sometimes struggle in their major [1] because they face difficulties in integrating large amount of knowledge into novel circumstances. Earlier, it was believed that studying medicine or medicine-related fields simply involved memorizing extensive information, and students who were able to memorize well were considered excellent students. In this regard, educators paid more attention to lectures because they are the most effective ways of delivering vast amounts of knowledge to students. However, owing to the rapid development of technology, students can access information and data more easily than ever before. Thus, amassing knowledge should not be the objective of learning anymore. Instead, students should be able to apply what they have learned to the problems at hand. Moreover, it has been found that traditional lecture-centered education does not lead to students’ effective learning [2]. Lectures fail to promote students’ thinking [3], and even extraordinary lectures by exceptional professionals cannot guarantee students’ performance [4].

Accordingly, flipped learning (FL) has emerged as an alternative to lectures. Nowadays, FL has been widely used in universities due to its potential to replace lecture-centered learning; however, it lacks a consistent effect. Therefore, as an effective alternative to a lecture, the active participation method in class has been explored multilaterally, a method commonly known as “active learning.” However, as the scope of active learning is broad, previous research have specified this concept into the Interactive-Constructive-Active-Passive (ICAP) framework [5, 6].

The ICAP framework describes four modes of cognitive engagment in active learning: Interactive, Constructive, Active, and Passive. The passive mode generally refers to sitting in listening to lectures, while in active modes students not only learn new knowledge but also physically manipulate the information learned. In constructive modes, students make greater effort to learn knowledge by drawing diagams or asking questions, rather than simply relying on the education materials. In interactive modes, two or more colleagues cooperate and co-construct through the process of asking questions and responding to one another during a conversation. The aforementioned classification and previous literature [6] confirmed that learning achievement is lowest at P and increases in the order of A, C, and I. Given that the ICAP framework involves both interactive and active learning, its application to the education of health professions would further promote and expand learning performance with regard to acquiring knowledge when comapred with FL, which only comprises active learning.

Not only are discussions more effective than listenig to lectures, which are more passive forms of learning, but previous research has also revealed that the quality of the dicussions may differ depending on what learning activity comes before them [7]. The quality of the dialogues increased when self-studying preceded the discussions due to the positive effect active learning had on memory and transfer of knowledge. In this study, we intend to establish an empirical learning model based on the ICAP framework that poses deeper learning will be promoted if the students are active. Hence, this study aims to identify the effectiveness of active learning based on the ICAP framework and its impact on the students' performance. Expanding previous studies, this study set active learning as two conditions: self-studying, and generating questions.

## Methods

### Participants

We conducted a priori power analysis using G*Power software to calculate a sufficient sample size to verify the effect of our main interest [8]. The power to detect a medium-sized effect (*f* = 0.25; cf. Cohen, 1977) was determined to be 0.4. Accordingly, participants were recruited at the Seoul National University’s undergraduate courses: 42 from medicine, 39 from dentistry, 36 from veterinary medicine, and 12 from nursing. Among the 129 individuals, 61 were female. However, we excluded participants who scored 5 points or more on the Likert scale for background knowledge questionnaires, indicating that these participants already possessed sufficient knowledge on the topic. Accordingly, 21 people were excluded from the experiment, and the data for only the remaining 108 participants were analyzed. (M_age_ = 19.58, SD_age_ = 1.04).

### Experiment procedure

Participants were asked to complete a questionnaire to report their background knowledge and interest. The participants were then instructed to study by themselves or attend a lecture. They studied a 7-page-long material. Participants were randomly assigned to four different study conditions: “lecture” or “self-study.” For the “self-study” group, participants were instructed to study written materials by themselves for 18 min. For the “lecture” group, participants were instructed to listen to a lecture while looking at written materials for 18 min. Subsequently, they were assigned to different question conditions: “question” or “summary.” They developed questions about the learning materials by themselves or simply summarized the materials prior to the discussion. The “question” group was asked to generate three questions in 5 min based on what they studied. The “summary” group had to summarize materials in three sentences in 5 min without generating questions. They discussed the questions they generated or that were formed by their peers, and finally, they were given 20 min to complete a posttest questionnaire. Additionally, each participant’s mental effort was measured for three times: after the study period, after the discussion, and after they completed the posttest.

Depending on our experimental design, participants were randomly assigned to each of the four groups: two study conditions (self-study vs. lecture) and two question conditions (question vs. summary). Specifically, there were four groups: [1] the lecture and question (LQ) group, [2] the lecture and summary (LS) group, [3] the self-study and question (SQ) group, and [4] the self-study and summary (SS) group.

### Questionnaires to check background knowledge

We used questionnaires to check participants' prior knowledge and interests to minimize the effects of these factors. Specifically, six questions were assessed using a 7-point Likert scale that include two topic-related questions. Participants were instructed to check if they possessed too much knowledge of the certain topics. They checked their depth of their knowledge depending on the 7-point scale ranging from “do not know about it at all [1]” to “know very well about it [7].”

### Posttest scores

Questions on the posttest were comprised of rote-memory type and transfer type items. The rote-memory type items consisted of ten questions testing facts directly from the given materials with a total of ten points. The four transfer type items required not only a full comprehension. These require a total comprehension of the given materials, total of 15 points. Thus, the total posttest score was scored out of possible 25 points.

### Mental effort measurement

In addition to our main experimental design, we measured the participants’ subjective mental effort for each study session (studying by oneself or listening to a lecture), discussion session (generating questions or writing a summary before the discussion), and taking the test. We checked whether mental effort spent by the participants on each learning activity significantly affected the learning outcomes of various groups. We also identified differences between the degree of mental effort spent in each condition and the participants’ major, leading to general conclusions regarding the patterns and relationships between learning and mental effort.

**Fig. 1:**
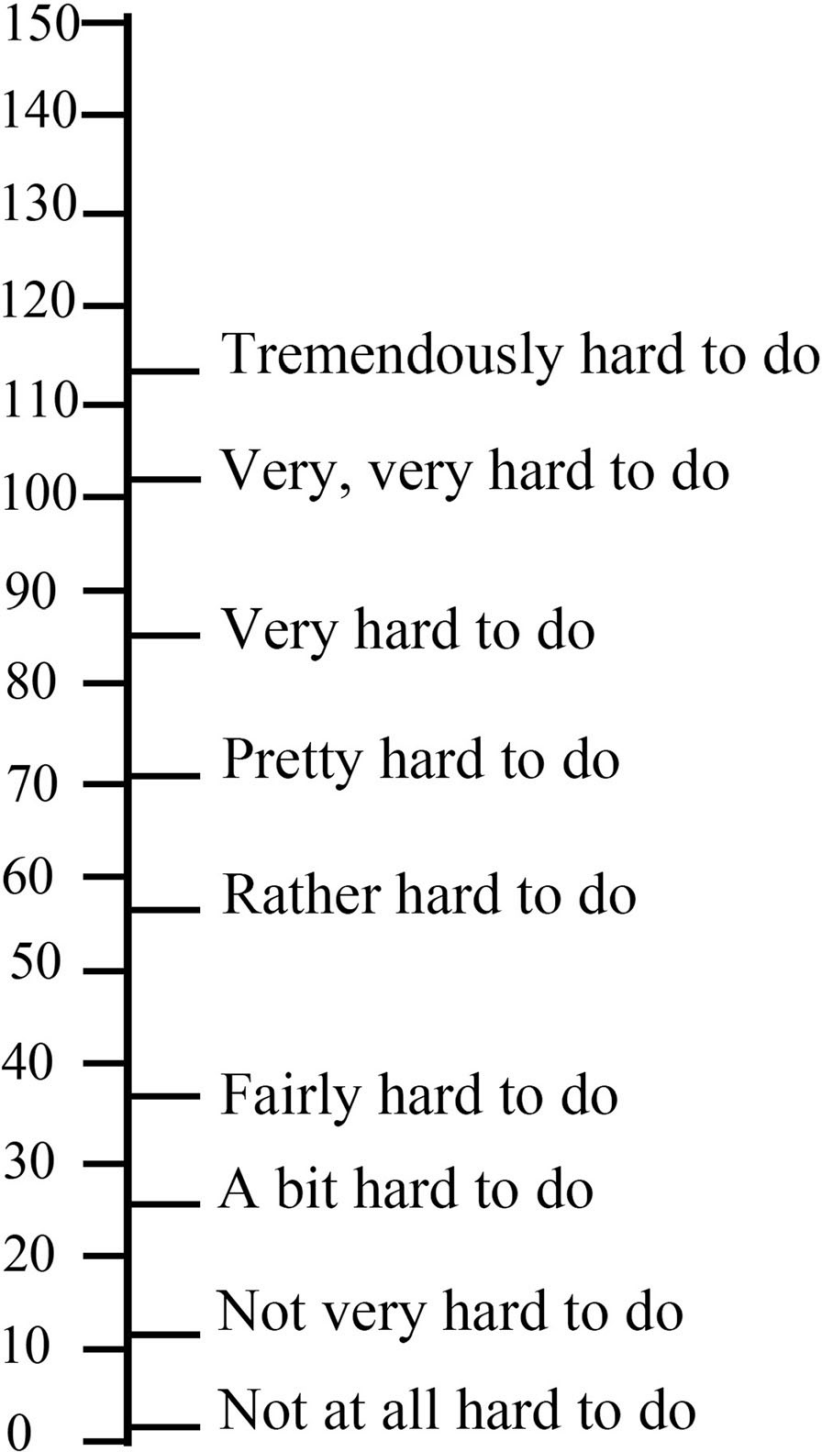
Subjective mental effort questionnaire

Participants were asked to fill out a Subjective Mental Effort Questionnaire after completing each session [7], i.e., after studying, after discussing, and after taking the test. The exact instructions required the participants to report the amount of cognitive pressure (effort) one felt during learning (discussing or testing) on a scale of 0–150. They were free to choose from any number on the left scale ascending in tens or from one of the expressions on the right (Fig. 1).

### Statistical analysis

To examine the effects of study conditions on learning outcome, a 3 × 4 mixed-design analysis of variance (ANOVA) was performed. In turn, the simple effect analyses using Bonferroni corrections were performed to verify the difference between study conditions. Bivariate correlation analyses were performed to explore the relationship between study variables and individual outcomes. Additionally,  mediation analysis was performed with 5000 bootstrapped samples to estimate the indirect effect among the main variables [9]. All statistical analyses were performed using SPSS 23 software (SPSS, Chicago, L, USA). The statistical significance for all tests was set as α <  0.05.

## Results

### Comparisons in learning achievement among conditions

Regardless of what group they were in, all participants studied the given materials for background knowledge. The materials used in this study are law-related and deal with the accusation, charge, and recognition of a criminal procedure code. The topic was chosen because it appeared less likely to be affected by background knowledge, and it seemed clear to set objective grading standards for posttest questions. Specifically, it was an unfamiliar topic for medicine-related students, and it had definite answers for transfer items to evaluate students’ performance. There were no significant age and background differences among the four groups.

Analyses were performed to compare the effect of study condition on learning outcomes. The results revealed a significant interaction effects of the study condition and learning outcomes (*P* <  0.001, Table 1). To diagnose this interaction effect at each level of sample, simple effect tests using Bonferroni comparisons were further performed. For total scores, SQ group performed better than the LQ and LS groups (*P* = 0.001; *P* <  0.001, respectively) but not the SS group (*P* = 0.827). The SS group had better performance than the LS group (*P* = 0.005). There was no significant difference between the LQ and LS groups. For rote-memory type item scores, the SQ and SS groups performed better than the LQ and LS groups (Ps <  0.05). However, there was no significant difference either between the SQ and SS groups or between the LQ and LS groups. Lastly, for transfer type item scores, the SQ group performed better than the LQ and LS groups (*P* = 0.003; *P* <  0.001, respectively) but not better than the SS group. However, there was no significant difference among the LQ, LS, and SS groups. The main effect of learning conditions was significant (Ps <  0.001), showing the same patterns as the result for transfer item scores in pairwise comparisons (SQ > LQ, LS). For item score type, the main effect was also significant, indicating the rote memory score was higher than transfer item score.

**Table 1:** Learning Performance according to type of items and study conditions

Type of items	LQ (*n* = 21)	SQ (*n* = 32)	LS (*n* = 29)	SS (*n* = 26)
Total score (25 points)	13.14 (3.61)	17.19 (3.75)	12.52 (2.79)	15.82 (3.56)
Rote memory (10 points)	7.33 (1.53)	8.47 (1.52)	7.38 (1.50)	8.70 (1.26)
Transfer (15 points)	5.81 (2.66)	8.72 (3.11)	5.14 (1.98)	7.12 (2.96)
ANOVA results	*F*	*P-value*	η^2^_p_	
Types of items (A)	10.471	< 0.001	0.112	
Study condition (B)	11.168	< 0.001	0.247	
Interaction term (A × B)	7.022	< 0.001	0.171	

Data are shown as mean (standard deviation). *LQ* Lecture and question, *SQ* Self-study and question, *LS* Lecture and summary, and *SS* Self-study and summary. For each LQ, SQ, LS, and SS group, total scores, rote-memory type item scores, and transfer type item scores are given. Gender and age were adjusted

### Mental effort invested in learning conditions

Within the analyzed data of 108 students, we excluded one participant who did not complete the questionnaire. The mental effort results were coded in an increasing level of difficulty as values of 0 (“Not at all hard to do”), 10, 25, 35, 55, 70, 85, 100, and 115 (“Tremendously hard to do”). ANOVA was performed to examine the differences in the degree of mental effort between different learning conditions.

Results showed that testing was the most difficult part of the learning session when compared with studying and discussing (mean mental effort: 67.30, 40.50, and 49.90, respectively, *P* < 0.001). Further differences in mental effort are shown in Table 2, where we checked whether different learning conditions (self-studying or attending a lecture, and having a discussion with self-created or given questions) significantly affected the degree of mental effort throughout the learning process. Although the degree of mental effort of participants of each study condition seemed to differ significantly, no significant difference was found between participants of the two question conditions. Specifically, participants who studied the learning material on their own displayed a higher mental effort during study periods (46.3 vs. 33.8, *P* = 0.009) but marginally lower degrees while taking the test (62.6 vs. 72.7, *P* = 0.061), compared with those who watched a lecture.

**Table 2:** Differences in mental effort by learning conditions

Variables	Study conditions (*n* = 108)				
	Lecture (*n* = 50)	Self-study (*n* = 58)	F	η^2^_p_	*P-value*
M1	33.80 (24.19)	46.32 (25.21)	6.984	0.063	0.009
M2	46.20 (31.01)	53.16 (29.89)	1.505	0.014	0.223
M3	72.70 (23.11)	62.63 (28.40)	3.594	0.034	0.061
Variables		Question conditions (n = 108)			
	Summary (*n* = 55)	Question (*n* = 53)	F	η^2^_p_	*P-value*
M1	43.49 (24.82)	37.50 (25.84)	1.212	0.274	0.274
M2	47.55 (28.53)	52.22 (32.37)	0.428	0.515	0.515
M3	69.53 (25.33)	65.19 (27.52)	0.673	0.414	0.414

Data are shown as mean (standard deviation). M1 Study mental effort, M2 Discussion mental effort, M3 Test mental effort. Gender and age were adjusted

### Impact of mental effort on learning outcome

The overall impact of mental effort on learning outcome can be seen from the correlation analysis between each mental effort and test performance scores (Table 3)*.* From the results, it was confirmed that only testing mental effort showed a weak negative correlation with total score (*r* = − 0.37, *P* < 0.001). In addition, participants who showed a higher level of testing mental effort also showed higher levels of studying and discussion mental effort (*r* = 0.43, *P* < 0.001; and *r* = 0.38, *P* < 0.001, respectively).

**Table 3:** Correlations between study variables

Variables	Total score	Scores of transfer type items	Scores of rote-memory type items	M1	M2	M3
Total score	1.00					
Scores of transfer type items	0.93^***^	1.00				
Scores of rote-memory type items	0.70^***^	0.39^***^	1.00			
M1	.00	−0.10	0.05	1.00		
M2	−0.40	0.03	−0.06	0.43^***^	1.00	
M3	−0.37^***^	−0.36^***^	−0.29^**^	0.35^**^	0.38^***^	1.00

*M1* Study mental effort, *M2* Discussion mental effort, *M3* Test mental effort. ^**^P < 0.01, ^***^P < 0.001

### Simple mediation analysis

**Fig. 2:**
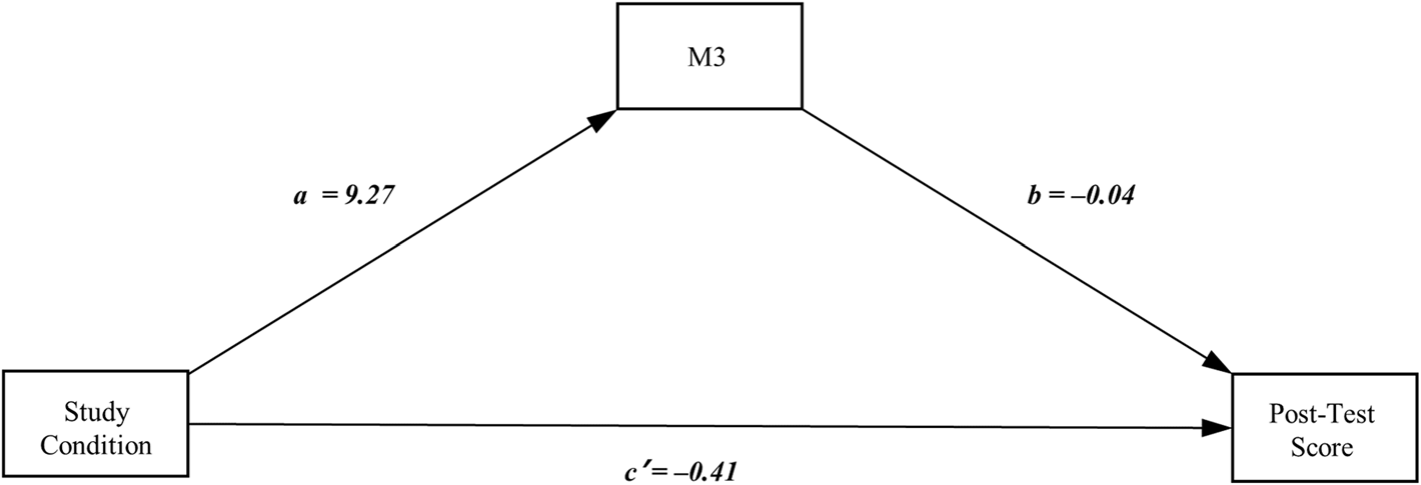
Simple mediation model (path a, association between study conditions and mental effort; path b, association between mental effort and test performance; and path c’, direct effect between study conditions and test performance). Gender and age were adjusted. M3 = Test mental effort. ^*^P < 0.05, ^***^P < 0.001

Based on correlation results and group differences above, a simple mediation model (Fig. 2) was tested to confirm our hypothesis that mental effort would mediate the effect of different learning conditions on learning outcomes. A simple mediation model was run separately for each mental effort (i.e. studying, discussing, and testing), different study conditions (studying by oneself or listening to a lecture) and discussion conditions (generating questions or writing a summary). Learning outcome in this model refers to the total posttest score. To test for mediation effect, a bootstrapping method using 5000 bootstrap samples and 95% bias-corrected confidence intervals (CIs) was performed, adjusting gender and age as covariates. The results were considered significant when CIs did not include 0.

Among mental effort variables, only the test mental effort (M3) showed a significant negative indirect effect which was significantly different from zero (b = − 0.41; standard error [SE] = 0.24; 95% bootstrap CI = − 0.9599 to − 0.0018). The direct effect of study condition on post-test score was also significant (b = − 3.30; SE = 0.66; 95% bootstrap CI = − 4.6076 to − 1.9948). These results indicated that the relationship between different study conditions and post-test performance was mediated by mental effort spent during the test.

## Discussion

Traditionally, medical education has been focused on how to teach facts and knowledge for students to learn; therefore, lecturer-centered education was considered the best way to learn. However, over the past decades, there have been many changes in medical education, and various interventions have been tried and tested to enhance students' learning outcomes [10]. This educational trend has also emerged in medicine-related fields, resulting in strategies for active learning that includes Problem-Based Learning (PBL) [11]. Active learning is learner-centered, where individual’s needs are considered more essential than those of the group [12]. In that regard, a learner needs to not only learn actively about given tasks but also reflect on what they are studying. Active learning supposes that knowledge can be obtained by a learner oneself, whereas passive learning assumes that knowledge can be conveyed from one person (lecturer) to another (learner) [2]. As an example of active learning, PBL reflects that knowledge is constructed rather than received, for it is based on the assumption that knowledge arises from thinking about an authentic problem. While PBL has been successfully implemented in the curriculum for health professions, there are still several limitations compared to other collaborative learning methods [13].

As an alternative to PBL, Team-based learning (TBL) has gained recent popularity in medical education for health professions [14]. In contrast to PBL, TBL does not require several instructors but maintains the advantages of small group teaching and learning. This advantage of TBL allows instructer in health professions to provide learners with effective resources and a reliable environment for teams to deal with clinical problems in the real world [15]. In line with that reason, a previous study showed that students preferred TBL over PBL as the optimal teaching strategy in medical education [16]. However, as with other teaching methods, TBL also has some practical difficulties and limitations: initial time needed on the part of the lecturers (e.g., readiness of application exercises), probability of strong opposition from current policy makers, and sufficient physical classroom space needed compared with traditional education settings. Thus, the demand for a novel paradigm besides PBL and TBL applied to education for health professions has arisen.

To overcome constraints in PBL and TBL, medical education for health professions may benefit from applying the ICAP framework. According to our results, participants performed better in the self-study condition than lecture condition across the following types of items (Table 1): total, rote-memory, and transfer. The results suggest that rather than making learners passively listen to lectures, it can be more effective to ask them to engage with learning materials by themselves in education programs for health professions. The implication of our result is similar to a recently emerging educational concept, Flipped learning. In comparison with conventional learning, FL is distinctive in that the teacher gives a lecture on a certain topic before class to utilizes time for more active learning activities. As information technology advances, educational materials have become easily accessible and not just limited to classrooms (e.g., massive open online courses: MOOCs). Students can be actively involved in constructing knowledge in their own way, unbound to physical classrooms. In this regard, the role of actual classroom settings and lecturers, which were the primary source of information, should be transformed. Rather, students could gain a deep understanding of knowledge through activities such as concept exploration, meaning-making, experiential engagement, and demonstration/application during class time. Along with FL, our experimental results based on the ICAP framework may present practical implications and theoretical support for new pedagogical approaches.

According to many studies, including the ICAP framework, the learning outcome is greater when students participate in learning more actively. However, studies on how to maximize such interaction are difficult to find. Thus in this study, prior to having a discussion, a comparison between listening a lecture, and self-studying was made, and furthermore, the activities of simply receiving questions and generating questions were considered. As a result, the SQ group had the highest performance, and the LS group had the lowest performance when comparing with the total score of the posttest questionnaires. For rote-memory type item scores, the SQ and SS groups had higher performance than the lecture groups (LQ and LS). Finally, for transfer type item scores, the SQ group had the highest performance. In summary, students who studied by themselves and completed question-generating activities showed better performance compared to other students. This suggests that students who participate in more active learning activities rather than simply listening to lectures and receiving given questions experienced increased learning outcomes.

In addition to self-study, question generation can be a useful strategy in actively constructing knowledge for learners. The SS group showed higher performance in rote-memory items than the LQ/LS group, but there was no significant difference in performance on transfer items (Table 1). Only the SS group showed higher scores in comparison to the LQ/LS group in the transfer type items. These results suggest that not only summarizing what they have studied but also generating questions regarding the content can be effective in applying existing knowledge to other contexts. The students writing the questions for discussion themselves is more effective for learning than merely responding to given discussion questions [17]. When writing questions for the discussion, learners need to understand the given materials and through a process for “generating” new concepts based on prior knowledge. Therefore, learners need a higher level of thought than when they are given the discussion questions by others. Specifically, a discussion beginning with questions constructed by the learners can be more productive [18], improving the quality of the class by creating a rich discussion. During the discussion, learners may experience the knowledge-change process from interactive activity, which promoted transfer learning through sufficient understanding regarding the contents.

Among the two experimental treatments during study and discussion, only different study conditions showed significant results in the analysis of mental effort (Table 2). Based on the results of the analysis of mental effort, we found that the degree of testing mental effort significantly correlates with students’ learning outcomes (i.e., significant negative correlation between testing mental effort and posttest performance) (Table 3). Meanwhile results from the mediation analysis (Fig. 2) supported the previous study [19]. Overall, the difference in mental effort patterns between two studying conditions and the results of simple mediation analysis combined suggest how students’ study affects the degree of mental effort he or she spends on studying and testing, eventually influencing their learning performance [20]. The negative correlation can be interpreted as students spending unproductive effort during testing compared with when they study or discuss the materials. In other words, unlike effort spent in studying and discussing, it is too late for the effort spent taking the test to contribute to an increase in performance. It is possible that students who participated in self-study and question generation put in extra effort than those who listened to a lecture and wrote a summary, leading to a better understanding of the material and thus, higher performance. This is consistent with previous findings that support the elaborative retrieval theory, which suggests why learners must invest substantial mental effort during studying [19, 20].

Although, discussions themselves were not the focus of the current study, the results represent that self-study, a more active form of learning, was associated with the level of engagement during discussion. The mental effort in the study session (M2) positively correlated with the mental effort in the discussion session (M3), suggesting that learners who actively engage in self-study would also actively engage in the course of the discussion (Table 3). These results suggest that both the act of self-studying before class and the amount of mental effort during self-studying are important elements for active learning.

However, this study has some potential limitations. First, in the video used in this experiment, as with any online lectures, appears one lecturer [21, 22]. However, there can be many styles of lecture, one in which the professor interacts with the students when explaining a concept. Since different lecture styles may affect students’ performance, further experiments should incorporate different forms of lectures. Second, we measured mental effort after studying, discussion, and test-taking respectively. Accordingly, mental effort measured after the discussion or taking the test could have been affected by prior learning activities. This implies mental effort scores may not represent the pure cognitive load spent in individual learning activities. Nevertheless, our study’s purpose was to identify the effects of different prior learning activities on final academic performance. Following how learning performance, the study’s main dependent variable, is an accumulation of previous events, mental effort was intended to be studied in the same nature. For future research, to address the limitations of measuring mental effort, coding and further analysis of discussion dialogs could reveal how prior learning activities affect the quality of discussion and the amount of mental effort required accordingly. Lastly, in this experiment there were no interventions from the professor during the discussions. While the enthusiasm of the instructor may affect students’ performance [23], it seems necessary to include instructional interventions when experimenting with discussions in the future. This is because timely interventions can improve the quality of the dialogues and thus, positively affect learning outcome.

## Conclusion

Combined together, our findings demonstrate that it is better for learners to discuss after self-studying than listening to lectures. Moreover, creating their own discussion questions can maximize the learning outcomes. Overall, encouraging learners to participate actively in learning activities is important and can lead to an increase in the transfer of knowledge. Applying these findings to the curriculum will help improve medical education in health professions. The ICAP framework is expected to be useful in developing health professionals who should cope with new difficult situations by encouraging them to become active learners.

## Data Availability

The datasets used and/or analyzed during the current study are available from the corresponding author on reasonable request.

## Abbreviations

(LQ) group: Lecture and question group
(LS) group: Lecture and summary group
(SQ) group: Self-study and question group
(SS) group: Self-study and summary group
FL: Flipped learning
ICAP: Interactive-constructive-active-passive
PBL: Problem-based learning
TBL: Team-based learning
